# Multipoint Energy-Balanced Laser-Ultrasonic Transducer Based on a Thin-Cladding Fiber

**DOI:** 10.3390/s24051491

**Published:** 2024-02-25

**Authors:** Shengnan Zhou, Cheng Zhou, Jiajun Tian, Yong Yao

**Affiliations:** 1 School of Electronic and Information Engineering, Harbin Institute of Technology, Shenzhen 518055, China; 20b952006@stu.hit.edu.cn (S.Z.); 20b352001@stu.hit.edu.cn (C.Z.); yaoyong@hit.edu.cn (Y.Y.); 2 Zhengzhou Research Institute, Harbin Institute of Technology, Zhengzhou 450003, China

**Keywords:** ultrasonic transducer, fiber-optic, thin-cladding fiber, photoacoustic material, structural health monitoring

## Abstract

This study proposes a novel multipoint transducer system by utilizing the single-mode-multimode-thin-cladding fiber (SMTC) structure. This structure leverages the disparity in mode field diameter between the multimode fiber (MMF) and thin-cladding fiber (TCF) to generate high-amplitude ultrasonic signals safely and efficiently. The fabricated transducer exhibits signal amplitudes 2–3-fold higher compared to conventional laser-ultrasonic transducers. Simulation analysis investigates the impact of the length of the MMF and the diameter of the TCF on coupling efficiency. The coupling efficiency of individual transducer units can be accurately controlled by adjusting the length of the MMF. A three-point energy-balanced laser-ultrasonic transducer system was achieved, with improved energy conversion efficiencies, and the optimal thickness of candle soot nanoparticles (CSNPs) is experimentally determined. Additionally, we carried out experiments to compare the performance of the proposed SMTC-based transducer system under different material conditions using two different photoacoustic materials: graphite–epoxy resin and candle soot nanoparticle–polydimethylsiloxane (CSNP–PDMS) composite. CSNPs, as a cost-effective and easy-to-prepare composite material, exhibit higher photoacoustic conversion efficiency compared to graphite–epoxy resin. The proposed system demonstrates the potential for applications in non-destructive testing techniques.

## 1. Introduction

Active ultrasonic testing (AUT) is a non-destructive testing technique widely employed for inspecting the structural integrity of materials, particularly metals and composites, using ultrasonic waves [1,2]. Despite its effectiveness, AUT is known to require bulky piezoelectric (PZT) ultrasonic transducers and specialized equipment, resulting in higher costs compared to alternative testing methods. An alternative option is the utilization of fiber-optic laser-ultrasonic transducers, which employ optical fibers as the transduction medium. These transducers offer numerous advantages, including immunity to electromagnetic interference, and compatibility with diverse materials and environments [3,4,5]. Various types of fiber-optic ultrasonic transducers have been proposed using different photoacoustic materials to improve the performance and extend their applications [6]. Graphite–epoxy resin composites were among the earliest materials that were successfully used for ultrasonic excitation and exhibited a two-orders-of-magnitude improvement in conversion efficiency compared to metallic materials [7,8]. Polydimethylsiloxane (PDMS) is a commonly used polymer for optical ultrasound generation owing to its high bulk linear coefficient of thermal expansion [9]. Some composite elastic materials have also been used for ultrasonic wave excitation, including gold salt–PDMS nanocomposite [10], gold nanoparticles–PDMS nanocomposite [11,12], carbon black–PDMS composite [13,14], carbon nanotubes (CNT)–PDMS nanocomposite [15,16], multiwall carbon nanotubes (MWCNTs)–PDMS nanocomposite [17,18,19], and candle soot nanoparticle–polydimethylsiloxane (CSNP–PDMS) composite [20,21,22]. Among these materials, CSNP has gained significant attention as a straightforward method for fabricating carbon nanoparticles, primarily due to its hierarchical nanostructure nature [23]. However, the techniques involved, such as dip-coating, and the use of materials such as gold nanoparticle composites [11,12], CuInS_2_ quantum dots [24], and metal film [25] increase cost and system complexity; the photoacoustic conversion efficiency also needs improvement. Moreover, all these studies have been limited to single-point ultrasonic transducers. Previously, a multipoint fiber-optic sidewall transmitter was fabricated by polishing a section of the fiber cladding and subsequently replacing it with an absorbing coating [26,27]; however, a significant challenge was the precise control of energy coupling efficiency at each point, restricting the number of transducer units in the system. Tilted fiber Bragg gratings with distinct wavelength spectral modes were also used to achieve ultrasonic excitation by coupling specific spectral modes into cladding using a wavelength-scanning laser source [28]. However, this method required costly equipment and materials, and the number of transducer units was restricted by the bandwidth of the light source. Some ultrasonic generators achieve multipoint ultrasonic excitation through the design of different structures [29,30,31,32,33]; however, these designs have their limitations. The peanut-shaped structure [31] requires laser energy amplified to 1 W, which restricts the maximum number of transducer units; the fabrication of single-mode-coreless-single-mode fiber (SCS) structure requires hollow core fibers as fusion medium, which increases the complexity of the fabrication process [32]; the core-opened taper structure [33] faces challenges in controlling the coupling energy due to the simultaneous influence on the length and diameter of tapers during preparation. Therefore, to meet the growing demand for distributed applications, there is a pressing need to develop a new multipoint ultrasonic excitation scheme that is characterized by ease of preparation, cost-effectiveness, and enhanced energy conversion efficiency.

In this work, we propose a novel multipoint ultrasonic excitation system based on the single-mode-multimode-thin-cladding fiber (SMTC) structure. The theoretical analysis demonstrates that different transducer units with varying coupling efficiency can be prepared by adjusting the length of the multimode fiber, enabling multipoint ultrasonic excitation. In terms of photoacoustic materials, we compare the CSNP–PDMS composite and graphite–epoxy resin, and found that CSNPs offer higher photoacoustic conversion efficiency while being simple to prepare and cost-effective. Additionally, we experimentally determined the optimal thickness of the CSNPs and demonstrated a three-point ultrasonic transducer system. By optimizing the fiber structure, incorporating improved photoacoustic materials, and validating the system’s performance, the results of energy-balanced ultrasonic signals confirmed the system’s potential for distributed AUT.

## 2. Simulation and Fabrication

### 2.1. SMTC Structure

**Figure 1:**
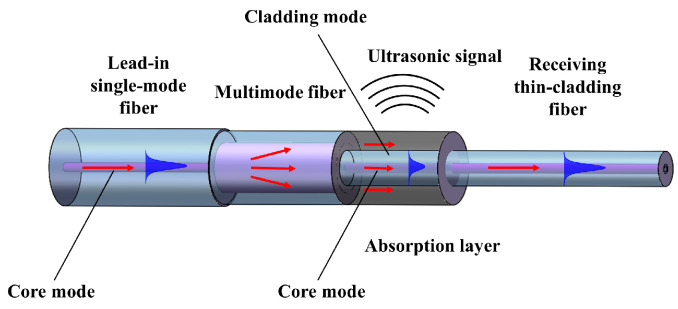
Schematic of the single-mode-multimode-thin-cladding fiber (SMTC) structure.

The SMTC structure, shown in Figure 1, comprises three components: a lead-in single-mode fiber (SMF), a multimode fiber (MMF), and a receiving thin-cladding fiber (TCF). The pulsed laser is transmitted through the lead-in SMF, and as it enters the MMF, the laser light is dispersed. The dispersed light propagates through the MMF, and as it reaches the interface of the TCF, a portion of light energy couples with the TCF core to form the fundamental mode, which is then transmitted through the receiving TCF. Another portion of light energy couples with the cladding to form high-order cladding modes, which then reach the light-absorbing layer of the photoacoustic material. Additionally, due to the disparity of the cladding thickness between the MMF and TCF, some of the light in the MMF cladding directly reaches the photoacoustic material and is then absorbed by the material. The periodicity of the pulsed laser causes the material to vibrate periodically, generating ultrasonic signals due to the thermoelastic effect. Consequently, compared to fibers with different diameters, TCF can yield a more substantial light absorption effect on photoacoustic material when absorbing the same light energy, resulting in an increased amplitude of the generated ultrasound. In previous studies [28,29,30,31,32,33], the etching process involved in the preparation greatly increases the risk of experimental operation and cannot guarantee the efficiency of the experiment. The TCF is used as a replacement for a cladding-etched fiber to enhance the absorption of light energy by the photoacoustic material, and generate stronger ultrasonic signals.

### 2.2. Transmission Mechanism within the SMTC Structure

Theoretical analysis of light wave propagation in the SMTC structure was performed using the BeamPROP module of the commercial software RSoft (2018) developed by Synopsys (Sunnyvale, CA, USA) for analyzing the dispersion properties of fibers. For the simulation, the initial source was defined as the fundamental mode of a SMF operating at a wavelength of 1550 nm, and a background refractive index of 1.0, which represents air, was assumed. A 1 mm long SMF was the lead-in fiber, and a 10 mm long TCF was the receiving fiber. The length of the MMF was 150 µm. Specific fiber parameters provided by the company were utilized in the simulation, including for the SMF (Corning (Corning, NY, USA), SMF-28, Ø125 µm), the core/cladding refractive index was 1.4521/1.4469, with a core diameter of 8.3 µm and a cladding diameter of 125 µm; for the MMF (YOFC (Wuhan, Hubei), SI2014-N, Ø125 µm), the core/cladding refractive index was 1.4446/1.4271, with a core diameter of 105 µm and a cladding diameter of 125 µm; the TCF (YOFC, BI1015-C, Ø60 µm), the core refractive index of was 1.4570, with a core diameter of 8.3 µm and a cladding diameter of 60 µm.

**Figure 2:**
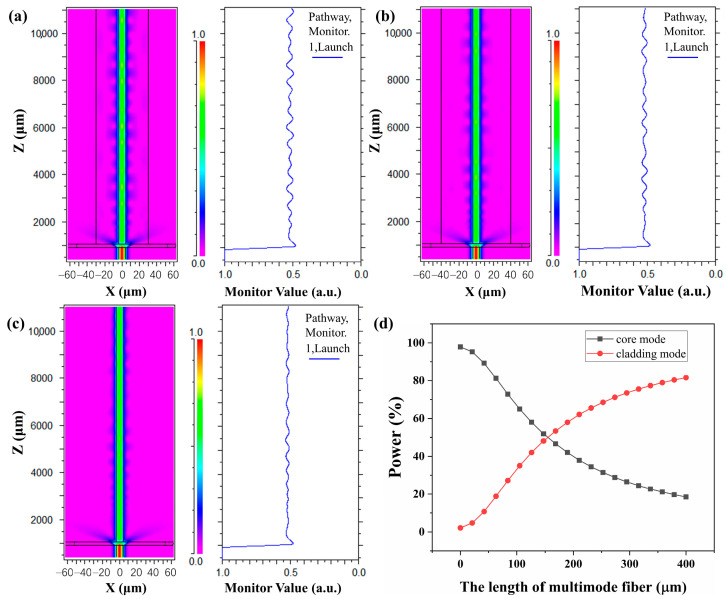
Transmitted light field distribution in the XZ plane and energy variation in the core mode through the SMTC structure with a 150 µm length of multimode fiber (MMF) for different receiving fiber diameters: (**a**) 60 µm, (**b**) 80 µm, and (**c**) 125 µm. (**d**) The relationship between MMF length and the power levels in the receiving thin-cladding fiber (TCF).

The transmitted light-field distribution results are shown in Figure 2a. The field distribution remained stable as the light propagated in the fundamental mode through the lead-in single-mode fiber (SMF) over a distance ranging from 0 to 1000 µm. As the light propagated toward the interface between the SMF and MMF, it dispersed and excited high-order modes due to the mismatch between the two fiber cores. The high-order modes then propagated within the MMF until they reached the interface between the MMF and TCF. At this point, due to the larger mode field diameter in the MMF compared to the TCF, some of the light energy was directly absorbed by the photoacoustic material for ultrasonic signal excitation, whereas the remaining light energy coupled into the TCF fiber core and cladding, generating high-order modes. The variation in light energy along the SMTC structure is shown on the left. The core-mode energy diffuses over the MMF region, resulting in a sharp decrease in the light energy, with approximately 50% of the remaining energy entering the receiving TCF. The light energy that coupled to the fiber core transformed into the fundamental mode of the TCF (typically LP01), whereas the light energy that coupled to the cladding forms the high-order mode (LP0m). However, the disparities in core diameter and mode field diameter between the MMF and TCF lead to a mode field mismatch, resulting in the local focusing of light and coupling of the multiple modes of the MMF into the core mode of the TCF. This interference between modes creates a small disturbance in the core-mode region, causing energy fluctuations in the core mode. We also conducted simulations for receiving fibers with diameters of 80 µm and 125 µm, as shown in Figure 2b,c. The results demonstrate that the diameter of the receiving fiber has minimal impact on the coupling efficiency of the structure. This finding is consistent with previous research that partially etching the fiber cladding does not affect the coupling ratio of the structure [28,29,30,31,32,33]. It is noteworthy that as the diameter of the receiving fiber increases, the mode field mismatch becomes smaller, resulting in a decrease in the energy fluctuations within the core region of the receiving fiber. However, the diameter difference between TCF and MMF makes the light absorption effect of photoacoustic materials greater, the smaller the diameter of the TCF, the more advantageous it is, but at the same time, the structure must still maintain mechanical strength and can be fused by the splicer easily. For these reasons, we adopted the 60 μm TCF. Figure 2d shows the simulated relationship between the MMF length and power levels in the receiving TCF. As the MMF length increases incrementally at a 20 μm interval from 0 to 400 µm, the power level in the fiber core mode decreases, whereas the power level of the cladding mode increases. This result highlights the direct correlation between the coupling efficiency of each SMTC structure and the length of the MMF. Consequently, adjusting the MMF length enables the development of SMTC structures with different levels of coupling efficiency, providing flexibility in tailoring the SMTC design to achieve desired performance characteristics.

### 2.3. Fabrication of SMTC Structure

**Figure 3:**
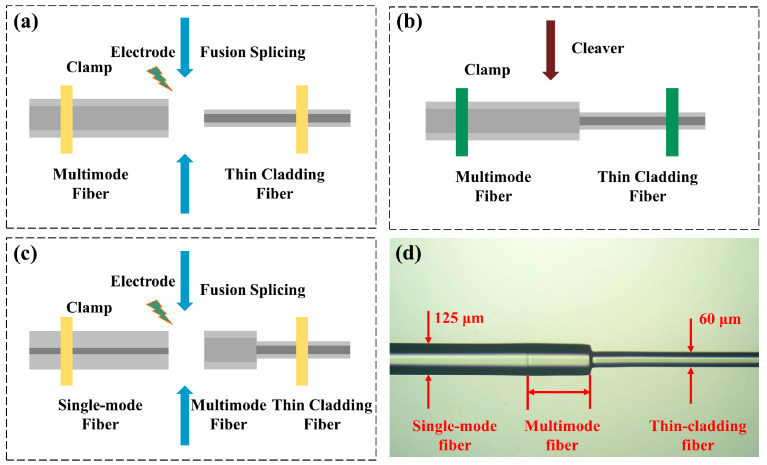
(**a**) Splicing the cleaved MMF with a TCF. (**b**) Cleaving the MMF to the desired length. (**c**) Splicing the fiber obtained in the (**b**) step with a single-mode fiber (SMF). (**d**) A microscopic image of the fabricated SMTC structure.

The fusion point of the MMF and TCF can be easily observed under a microscope due to disparities in their cladding sizes, avoiding the use of hollow core fiber as a fusion splicing medium, and simplifying their preparation process, particularly when the MMF is cleaved to the desired length. Figure 3a–c show the fabrication of the SMTC structure. First, the TCF and MMF were cleaved. Then, the two fibers were spliced using a fusion splicer (Fujikura FSM-80S (Tokyo, Japan)), as shown in Figure 3a. Next, the fused structure was placed on a displacement platform, and the platform was adjusted to obtain a desired MMF length facilitated by a microscope and cleaved, as depicted in Figure 3b. Finally, the fiber acquired from the previous step with the SMF was spliced, as shown in Figure 3c, successfully yielding the SMTC structure (Figure 3d). By performing these simple micro-scale cleaving and splicing steps, SMTC structures with the required length of the MMF can be fabricated according to the number of transducer units, thereby optimizing the system.

The coupling efficiency of SMTC structures can be measured using a laser source and an optical power meter in three straightforward steps. First, the output power of the light source is measured and recorded using the power meter. Then, the SMTC structure is spliced between the laser source and optical power meter, and the power meter value is re-recorded. Finally, the proportion of laser source energy coupled to the cladding in the SMTC structure is calculated by comparing the two recorded power meter values. Thus, the coupling efficiency of the SMTC structure can be accurately determined using this method, which is a crucial factor in optimizing its performance.

### 2.4. The Multipoint Ultrasonic Excitation System

**Figure 4:**
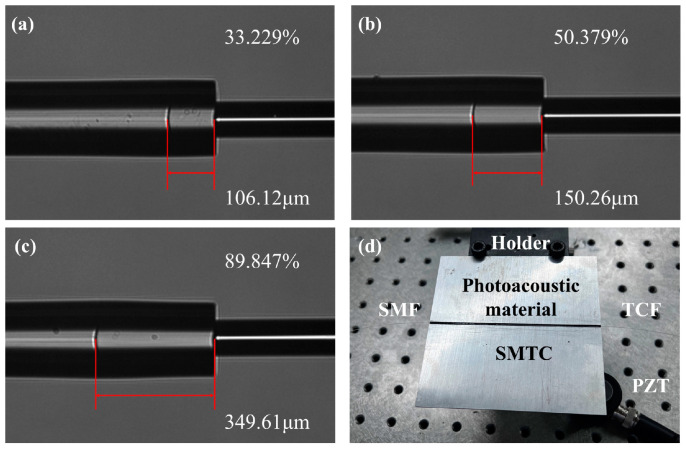
(**a**–**c**) Three SMTC structures with a coupling efficiency of 33.229%, 50.379%, and 89.847%. (**d**) Photograph of the ultrasonic transducer unit.

To ensure the uniform and stable generation of ultrasound signals from each SMTC-based transducer unit, precise energy balancing of the laser coupled to the fiber cladding is required. For instance, in a three-point ultrasonic transducer system, the energy consumption should be distributed equally among each transducer unit, with each unit receiving approximately 33% of the total energy. To achieve this, three SMTC structures were prepared with a coupling efficiency of 33.229%, 50.379%, and 89.847%, each with different MMF lengths of 106.12, 150.26, and 349.61 µm, respectively. Figure 4a–c show the microphotographs of the fabricated SMTC structures. The difference between the coupling efficiency noted in Figure 4c and the simulated values presented in Figure 2d is mainly attributed to the fusion splicing losses during the fabrication process. Therefore, the coupling efficiency values reported in this paper are based on precise measurements obtained in measurement. The distribution of extracted energy from the fiber core among the three structures is as follows: 33.229% in the first structure, 33.639% in the second, and 29.768% in the third. In the SMTC-based transducer system, the prepared fiber-optic SMTC structures are placed on a pre-fabricated aluminum plate measuring 100 × 100 × 1 mm^3^. The plate has a pre-etched groove at the center with a depth of 200 μm. Subsequently, the photoacoustic material is coated and heated for solidification. The configuration of the SMTC-based transducer unit is shown in Figure 4d. By connecting these SMTC-based transducer units in ascending order of coupling efficiency, a multipoint energy-balanced laser-ultrasonic transducer system can be achieved.

## 3. Experiment and Discussion

### 3.1. Experiment Setup

**Figure 5:**
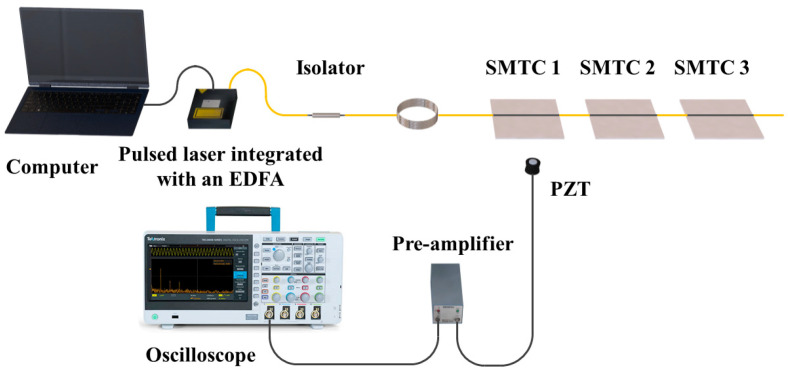
Experimental configuration for the multipoint energy-balanced laser-ultrasonic transducer system.

The effectiveness of the proposed system was experimentally verified. As shown in Figure 5, the experimental setup comprises a computer-controlled pulsed laser integrated with an EDFA amplification module (VLSS-1550-M-F-L-MP, CONNET (San Francisco, CA, USA)). The laser emits 5 ns, 3 kHz laser pulses. To safeguard the system, an isolator is employed to prevent any damage caused by the reflected light. The amplified pulsed laser sequentially reached each SMTC-based transducer unit, generating ultrasonic signals due to the thermoelastic effect of the photoacoustic material.

During the experiment, the ultrasonic signals were captured using a commercially available PZT transducer (V120-RB, OLYMPUS, Tokyo, Japan). The PZT transducer was carefully positioned beneath the aluminum plate to ensure accurate ultrasonic signal detection. A pre-amplifier (5660C, OLYMPUS) was used to amplify the ultrasonic signals by approximately 60 dB, and the amplified signals were easily observed and recorded for the detailed analysis of signal characteristics using an oscilloscope (MDO3104, TEKTRONIX, Beaverton, OR, USA).

### 3.2. Characterization of the Pulse Laser Light Source

**Figure 6:**
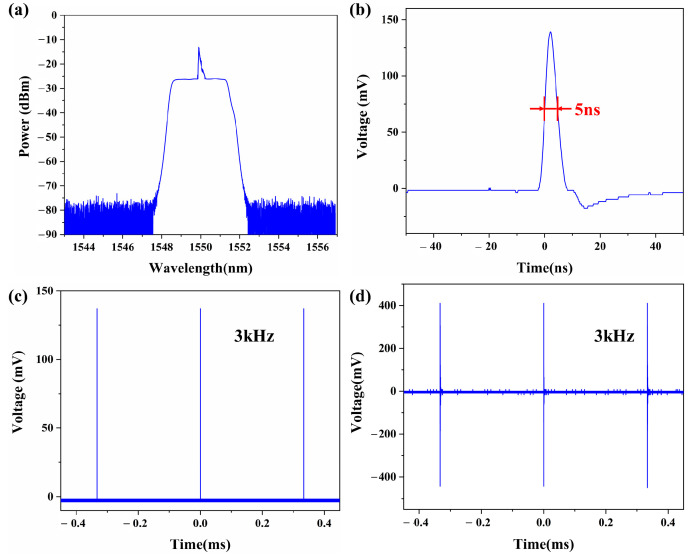
(**a**) Spectrum of the computer-controlled pulsed laser integrated with an EDFA amplification module measured by a spectrometer. (**b**) The single laser pulse with a 5 ns pulse width. (**c**) Pulse sequence with a with a 3 kHz repetition frequency. (**d**) The ultrasonic signal of the third SMTC structure with a 3 kHz repetition frequency.

The pulsed laser light source is a critical component in the effective operation of the ultrasonic transducer units; herein, its output performance was evaluated. A spectrometer (AQ6370C, YOKOGAWA, Tokyo, Japan) with a resolution of 0.02 nm was used to measure the spectrum of the light amplified by the EDFA. The pulsed laser parameters were set to a repetition rate of 3 kHz and a pulse width of 5 ns, consistently with the experimental settings, and the resulting spectrum is presented in Figure 6a, with a central wavelength of approximately 1549.9 nm and a corresponding bandwidth of 2.25 nm. To study the temporal characteristics of the transducer system, we used a photodetector (PDB410C, THORLABS, Newton, NJ, USA) to detect the output signal and recorded its waveform using an oscilloscope (DSOX3014T, KEYSIGHT, Santa Rosa, CA, USA). The recorded pulse width of a single pulse signal was 5 ns, with a maximum amplitude of approximately 150 mV, as shown in Figure 6b. The pulse sequence (Figure 6c) shows that the amplified laser from the EDFA exhibits a pulse interval of approximately 3.3 ms, which matches the repetition frequency of the pulsed laser at 3 kHz. Moreover, each pulse has nearly identical peak width and pulse power. The signal generated by the third SMTC structure is shown in Figure 6d, where each peak is excited with a nearly equal energy with a relaxation time of approximately 4 µs. The repetition rate of the ultrasonic signal remains constant, matching that of the pulse laser. These measurements have provided invaluable insights into the pulsed laser characteristics, which is essential for ensuring the optimal performance of this system.

### 3.3. Fabrication of Photoacoustic Material

The preparation method for transducers with graphite–epoxy resin was consistent with previous research [28,29,30,31,32,33]. Here, we primarily focus on the fabrication process of the transducers with CSNP–PDMS composite. CSNPs were synthesized through a flame synthesis method using a candle (diameter: 25 mm) composed of paraffin at room temperature [34]. When the candle flame stabilized, the flame height was approximately 3 cm. The groove of an aluminum plate was then placed 2 cm above the flame’s wick. Over a period of 30 s, a uniform layer of CSNPs covered the groove. Subsequently, a mixture of PDMS base material and curing agent (Dow Corning 184) was prepared, with a mass ratio of 10:1. Then, the mixture was coated onto the top of the CSNP-coated groove using a syringe, excess material was removed, and the sample was cured in an oven at 65 °C for 2 h.

**Figure 7:**
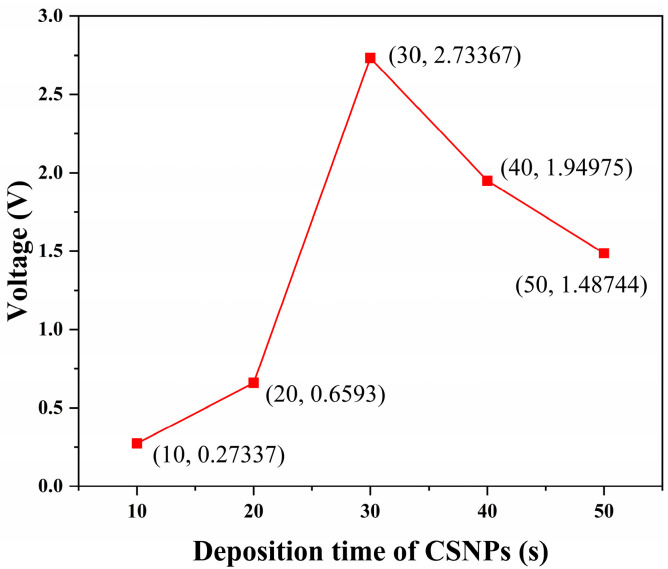
Amplitude of the generated ultrasonic signal changes with deposition time.

Photoacoustic materials consist of two layers: the light-absorbing layer and the thermal-expanding layer. The light-absorbing layer absorbs the energy of the excitation laser and converts it into heat, which is then transferred to the thermal-expanding layer, causing thermal expansion and ultimately generating ultrasonic signals. The conversion efficiency of photoacoustic effects is directly proportional to the optical absorption coefficient, which in turn is correlated with the concentration of the absorbing material; however, exceeding a certain thickness can reduce solidification efficiency [35,36]. Therefore, it is crucial to investigate the thickness of the absorbing material to optimize transducer performance. Considering the linear relationship between the deposition thickness of CSNP and deposition time [21,37], we fabricated five sets of SMTC structures with a constant coupling ratio of 50%. The amplitude of the generated ultrasonic signal was studied by varying deposition time. Figure 7 shows the results. As the deposition time of CSNPs increased, the amplitude of the ultrasonic signal also increased, reaching 2.73367 V at 30 s. However, further increase in deposition time resulted in a weakening of the ultrasonic signal amplitude due to the negative impact of excessive thickness on the curing process.

### 3.4. Results

The average power of computer-controlled pulsed laser integrated with an EDFA amplification module was tuned to 40 mW, resulting in a single pulse energy of 0.012 mJ; each SMTC-based transducer unit extracted approximately 0.004 mJ of energy. The photoacoustic material absorbed the coupled laser energy, producing periodic expansion and contraction and generating ultrasonic signals, which were measured using a PZT transducer.

**Figure 8:**
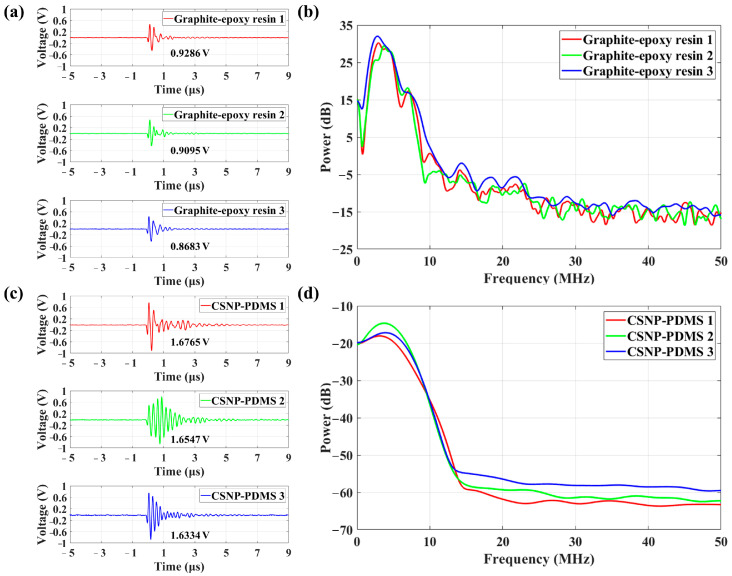
Ultrasonic signals excited by two three-point ultrasonic transducer systems: SMTC-based transducer system with (**a**) graphite–epoxy resin, (**c**) candle soot nanoparticle–polydimethylsiloxane (CSNP–PDMS) composite in time-domain, and (**b**,**d**) frequency analysis matched with the left.

To further optimize the system, we fabricated transducer units using the same ratio of graphite–epoxy resin as utilized in earlier studies [7,8,28,29,30,31,32,33], and SMTC-based transducer units with a coupling efficiency of 33.192%, 50.443%, and 89.864% using CSNP–PDMS composite. A control experiment was conducted under identical environmental conditions. The obtained time-domain and frequency-domain signals are shown in Figure 8. Figure 8a depicts the time-domain waveforms of each SMTC-based transducer units with graphite–epoxy resin, with peak-to-peak amplitudes measuring 0.9286 V, 0.9095 V, and 0.8683 V. These waveforms underwent fast Fourier transform (FFT) to obtain their corresponding frequency-domain spectra. The resulting frequency-domain signals exhibited a bandwidth of approximately 8.385 MHz, centered around 4.4 MHz (Figure 8b). In the frequency spectrum of the ultrasound signal, multiple frequency components are observed, indicating the existence of distinct reflection signals on the surface of the aluminum plate. Furthermore, the disparities in acoustic impedance values between graphite, epoxy resin, and aluminum plate lead to the reflection of ultrasonic waves at the interface during propagation, generating ultrasonic waves of varying frequencies [38]. As shown in Figure 8c, the peak-to-peak amplitudes of each SMTC-based transducer units using CSNP–PDMS composite in the time domain increased to 1.6765 V, 1.6547 V, and 1.6334 V. The resulting frequency-domain signal exhibited a broad bandwidth of approximately 11.7112 MHz, with a center frequency of around 3.3 MHz (Figure 8d). Notably, it displayed a distinct single-frequency component. As shown in Table 1, despite a significant relative error between measured values of SMTC3 in both systems and the theoretical value of 33%, the resulting peak-to-peak amplitudes remain within an acceptable range. These findings suggest that SMTC-based transducer units, utilizing two different photoacoustic materials, can achieve energy-balanced ultrasound excitation.

To comprehensively analyze the performance disparities of ultrasonic signals generated by SMTC-based transducer units with varying coupling efficiency and different photoacoustic materials, both time-domain and frequency-domain analyses were conducted. The relationship between the SMTC-based transducer units and their corresponding time-domain signals and frequency-domain signals is illustrated in Table 2, which includes parameters such as ultrasonic peak voltage, pulse duration, peak power, and bandwidth. In the time domain, for SMTC structures utilizing graphite and epoxy resin as the photoacoustic material, the peak-to-peak amplitudes of the signals were measured as 0.9286 V, 0.9095 V, and 0.8683 V, respectively. The average value was calculated as 0.9022 V, with a standard deviation of 0.025. The pulse durations were 1.8544 µs, 1.9776 µs, and 1.9800 µs, resulting in an average value of 1.9373 µs, with a standard deviation of 0.0585. For SMTC structures incorporating the CSNP–PDMS composite as the photoacoustic material, the peak-to-peak amplitudes of the signals were measured as 1.6765 V, 1.6547 V, and 1.6334 V, respectively. The average value was found to be 1.6549 V, with a standard deviation of 0.0174. The pulse durations were 5.3418 µs, 5.7364 µs, and 5.1710 µs, resulting in an average value of 5.4164 µs, with a standard deviation of 0.2249. Upon comparison of the results, it becomes apparent that the ultrasonic signals generated by the CSNP–PDMS composite exhibit a significant increase in peak-to-peak amplitudes, accompanied by a relatively smaller standard deviation. However, the pulse widths of the ultrasonic signals generated by the graphite and epoxy resin were smaller, indicating the presence of higher-frequency components. In Figure 8c, the wider pulse width in time-domain of the second transducer unit indicates that ultrasound waves underwent multiple reflections. This phenomenon may be attributed to the uneven deposition of candle soot, which serves as a light-absorbing material. To address this issue, it is advisable to ensure a more uniform deposition of candle soot for consistent and reliable results, such as the utilization of a higher precision-controlled robotic arm in future practical applications [39].

In the frequency domain, for SMTC structures utilizing graphite and epoxy resin as the photoacoustic material, the calculated standard deviation of the peak power was determined to be 0.7533 dB, with an average of 27.0013 dB. The standard deviation of the bandwidth was 0.2107 MHz, with an average of 8.3850 MHz. Similarly, for SMTC structures incorporating the CSNP–PDMS composite as the photoacoustic material, the calculated standard deviation of the peak power was found to be 1.0660 dB, with an average of −14.7935 dB. The standard deviation of the bandwidth was 0.3181 MHz, with an average of 11.7112 MHz. Notably, the CSNP–PDMS composite exhibited a broader bandwidth in the frequency domain for the generated ultrasonic signals. These results solidify the fact that the SMTC-based transducer unit demonstrates a high degree of stability in the frequency domain. The energy conversion efficiency was also calculated, as shown in Table 3, a comparison was made regarding the energy conversion efficiency of the transducer units used in the previous multipoint system [29,30,31,32,33]. It can be observed that the SMTC structure, due to its characteristics, exhibits higher energy conversion efficiency. Additionally, the transducer units prepared with CSNP–PDMS composite demonstrate a competitive advantage in terms of energy conversion efficiency.

**Figure 9:**
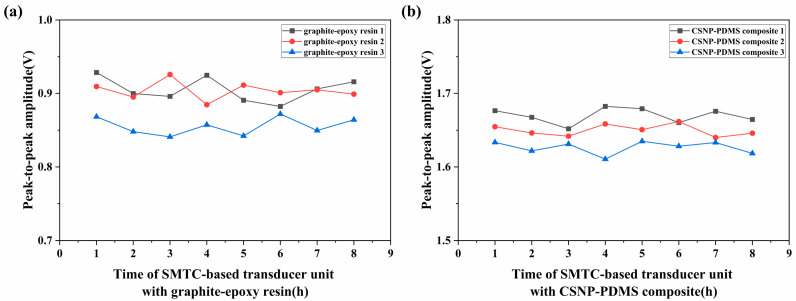
Evaluation of the stability of the ultrasonic signals produced by two systems.

The stability and reliability of the ultrasonic transducer units were evaluated in the same testing environment during an 8-h period, as shown in Figure 9. The obtained results clearly illustrate consistently small peak-to-peak amplitude fluctuations in the ultrasonic signals generated by the three transducer units. In Figure 9a for the SMTC-based transducer units with graphite–epoxy resin, the standard errors were found to be 0.01642, 0.01437, and 0.01103. Similarly, in Figure 9b for the SMTC-based transducer units employing the CSNP–PDMS composite, the standard errors were relatively more stable, measuring 0.005475, 0.004428, and 0.006435. These fluctuations were mainly attributed to variations in the power of the light source. Additionally, the fiber-optic ultrasonic transducer units exhibit high stability and reliability even after operating for long hours, without affecting the detection results.

## 4. Conclusions

In summary, a cost-effective fiber-optic ultrasonic transducer based on the SMTC structure was proposed herein and demonstrated for multipoint ultrasonic excitation. By applying the MMF and TCF, the need for hazardous and intricate etching processes on fiber cladding or fusion mediums is eliminated. The manufacturing process of the SMTC structure involves simple cleaving and splicing techniques, enabling safe and efficient generation of high-amplitude ultrasonic signals. Simulation results demonstrate that increasing the length of the multimode fiber leads to more laser energy being coupled into the cladding, while the diameter of the TCF does not affect the coupling efficiency. Therefore, different coupling efficiencies of SMTC transducer units can be achieved by adjusting the length of the multimode fiber. Furthermore, a comparison was conducted between two ultrasonic transducer systems, each composed of three transducer units fabricated using graphite–epoxy resin and CSNP–PDMS composite, respectively. Experimental results show that the multipoint laser ultrasound transducer system exhibits stable peak-to-peak amplitudes with average values of 0.9022 V and 1.6549 V, and energy conversion efficiencies of 225.55 mV/J and 413.725 mV/J, respectively. These performance and stability characteristics make this multipoint system a promising candidate for AUT applications.

## Figures and Tables

**Table 1:** Extracted energy of transducer units with theoretical and measured value.

Transducer Units	Theoretical Value (%)	Measured Value (%)	Relative Error (%)
Graphite–epoxy resin 1	33.333	33.229	−0.096
Graphite–epoxy resin 2	33.333	33.639	−0.005
Graphite–epoxy resin 3	33.333	29.768	3.474
CSNP–PDMS 1	33.333	33.192	−0.159
CSNP–PDMS 2	33.333	33.699	−0.016
CSNP–PDMS 3	33.333	29.753	3.535

**Table 2:** The generated ultrasound signals in time domain and frequency domain.

Transducer Units	Coupling Efficiency (%)	Peak-to-Peak Amplitude (V)	Pulse Width (μs)	Peak-to-Peak Power (dB)	−3 dB Bandwidth (MHz)
Graphite–epoxy resin 1	33.229	0.92864	1.8544	26.6610	8.5139
Graphite–epoxy resin 2	50.379	0.90951	1.9776	25.9005	7.9731
Graphite–epoxy resin 3	89.847	0.86834	1.9800	28.4425	8.6682
CSNP–PDMS 1	33.192	1.67650	5.3418	−16.2182	12.3013
CSNP–PDMS 2	50.443	1.65467	5.7364	−12.7076	11.6217
CSNP–PDMS 3	89.864	1.63344	5.1710	−15.4547	11.2105

**Table 3:** Comparison of the energy conversion efficiency of different multipoint systems.

Transducer Units	WET [29]	COS [30]	PNS [31]	SCS [32]	COT [33]	SMTC (Graphite–Epoxy Resin)	SMTC (CSNP–PDMS)
Laser pulse energy at each unit (mJ)	0.010	0.008	0.333	0.008	0.010	0.004	0.004
Average peak-to-peak amplitude (V)	0.54575	0.4995	0.5480	0.51260	0.49025	0.9022	1.6549
Energy conversion efficiency (mV/J)	54.575	62.4375	1.6455	64.075	49.025	225.55	413.725

## Data Availability

No new data were created or analyzed in this study. Data sharing is not applicable to this article.
